# Wave intensity analysis in the internal carotid artery of hypertensive subjects using phase-contrast MR angiography and preliminary assessment of the effect of vessel morphology on wave dynamics

**DOI:** 10.1088/1361-6579/aadfc5

**Published:** 2018-10-18

**Authors:** S Neumann, F Sophocleous, M D Kobetic, E C Hart, A K Nightingale, K H Parker, M K Hamilton, G Biglino

**Affiliations:** 1 University of Bristol, Bristol, United Kingdom; 2 University Hospitals Bristol NHS Foundation Trust, Bristol, United Kingdom; 3 Imperial College London, London, United Kingdom; Sandra.Neumann@Bristol.ac.uk

**Keywords:** wave intensity analysis, reservoir pressure, statistical shape modelling, internal carotid artery, blood pressure, hypertension

## Abstract

*Objective*: Hypertension is associated with reduced cerebral blood flow, but it is not known how this impacts on wave dynamics or potentially relates to arterial morphology. Given the location of the internal carotid artery (ICA) and risks associated with invasive measurements, wave dynamics in this artery have not been extensively assessed *in vivo*. This study explores the feasibility of studying wave dynamics in the internal carotid artery non-invasively. *Approach*: Normotensive, uncontrolled and controlled hypertensive participants were recruited (daytime ambulatory blood pressure  <135/85 mmHg and  >135/85 mmHg, respectively; *n*  =  38). Wave intensity, reservoir pressure and statistical shape analyses were performed on the right ICA and ascending aorta high-resolution phase-contrast magnetic resonance angiography data. *Main results*: Wave speed in the aorta was significantly lower in normotensive compared to hypertensive participants (6.7  ±  1.8 versus 11.2  ±  6.2 m s^−1^ for uncontrolled and 11.8  ±  4.6 m s^−1^ for controlled hypertensives, *p*  =  0.02), whilst there were no differences in wave speed in the ICA. There were no significant differences between the groups for the wave intensity or reservoir pressure. Interestingly, a significant association between the anatomy of the ICA and wave energy (FCW and size, *r*^2^  =  0.12, *p*  =  0.04) was found. *Significance*: This study shows it is feasible to study wave dynamics in the ICA non-invasively. Whilst changes in aortic wave speed confirmed an expected increase in arterial stiffness, this was not observed in the ICA. This might suggest a protective mechanism in the cerebral circulation, in conjunction with the effect of vessel tortuosity. Furthermore, it was observed that ICA shape correlated with wave energy but not wave speed.

## Introduction

Arterial wave transmission is of great interest to better understand integrated cardiovascular physiology. One method by which arterial wave transmission can be analysed is wave intensity analysis (WIA). Studies have shown that the WIA parameters in the ascending aorta are associated with cardiovascular function, whereby increased wave speed indicates increased vessel stiffness, the intensity of the forward compression wave (FCW) relates positively to myocardial contractility and the forward expansion wave (FEW) to diastolic relaxation of the heart (Ohte *et al* 2003). By definition ‘compression’ and ‘expansion’ refer to changes in the pressure, so that compression waves in the arterial system are associated with an expansion in the arterial diameter, whilst the expansion wave is associated with a reduction in diameter (Hughes *et al* 2008).

Traditionally, WIA has been performed on the basis of pressure/velocity data (Parker and Jones 1990). However, WIA can be adapted to allow for the assessment of wave reflections, energy transmission as well as deriving the pulse wave speed from non-invasive measures, such as phase contrast magnetic resonance (MR) imaging (Vulliémoz *et al* 2002, Li *et al* 2010, Biglino *et al* 2012). Phase contrast imaging allows high spatio-temporal resolution images of arterial flow in vessels of both large and medium calibre, e.g. the internal carotid artery (ICA). From a clinical standpoint, assessment of wave intensity in the internal carotids could be of interest given recent findings suggesting that increased blood pressure carries an increased risk of lacunar infarcts as well as a reduction in brain blood flow (Muller *et al* 2012, Warnert *et al* 2016).

One way the artery buffers the large pressure waves created during systole is by the slow discharge of blood volume during diastole. This is known as the reservoir, or Windkessel, effect. When measuring the reservoir pressure, an excess pressure can be found, defined as the difference between the measured reservoir and arterial pressures (Wang *et al* 2003). Parameters derived from the reservoir and excess pressure waveforms have been shown to correlate with both central and brachial blood pressure (Tyberg *et al* 2009, Narayan *et al* 2015). Little is known about the reservoir pressure of the internal carotid arteries given firstly the risks associated with invasive measurements, such as catheter-based pressure measurements, and secondly, given the position of the vessel making it less accessible with most non-invasive technologies. Previous studies have demonstrated the possibility of deriving reservoir/excess data from area and velocity obtained through CMR imaging (Gray *et al* 2016) and here we intend to further demonstrate the feasibility of analysing the reservoir/excess pressure from phase contrast data not only in the aorta but also in the ICA.

Lastly, it has been suggested that hypertension may be associated with increased incidence of abnormal arterial morphology and anatomy in the cerebral circulation such as vertebral artery hypoplasia and incomplete circle of Willis (Warnert *et al* 2016). Whilst vessel morphology can be assessed clinically, it is desirable to be able to quantify anatomical measures, such as the tortuosity of the vessels, to better understand how their structure may relate to function, especially in relation to their three-dimensional (3D) configuration. Therefore, we used a statistical shape modelling approach to assess the anatomy of the ICA (Bruse 2016, 2017).

The overall aim of this study was to assess the feasibility of WIA and reservoir analysis in the ICA and to explore whether these parameters relate to the morphology of the vessel. We specifically hypothesised that a greater systemic blood pressure would lead to greater wave speed, greater reservoir pressure integral and greater net wave energy in the ICA. We also expected that higher wave speed as an indicator of stiffening of the vessels would be associated with greater amplitude of the reflected backwards compression wave.

## Methods

This study was performed with approval from NHS UK research and ethics committee Exeter with reference 15/SW/0176. All methods conformed to the declaration of Helsinki.

### Participants

Participants (*n*  =  38) who entered into the study had their blood pressure measured over 24 h using an ambulatory monitor. Based on the daytime ambulatory blood pressure (dABPM), participants were classified as either *normotensive* (NTN, dABPM  <135/85 mmHg with no anti-hypertensive therapy, *n*  =  13), *uncontrolled hypertensive* (uHTN, dABPM  >135/85 mmHg with no treatment or despite anti-hypertensive medication, *n*  =  12) or *controlled hypertensive* (cHTN, dABPM  <135/85 mmHg with anti-hypertensive medication, *n*  =  13). Participants presenting with major illness leading to cardiovascular dysfunction such as diabetes, cardiovascular disease not directly associated with hypertension, neurological, renal, respiratory or immune disorders were excluded from the study. All participants were screened with a 12-lead ECG for cardiac abnormalities and a urine dipstick for symptoms of renal abnormalities (Siemens multistix).

### Image acquisition

A time of flight (ToF) angiogram of the cerebral feeding arteries and the circle of Willis was acquired for the positioning of the flow slices intra-cerebrally and for the 3D modelling of the artery shape (echo time (TE)  =  7.0 ms, repetition time (TR)  =  23 ms, flip angle  =  25°, averages  =  1, voxel size  =  0.8  ×  0.8  ×  0.8 mm, field of view  =  200 mm, matrix  =  256  ×  100 pixels).

**Figure 1.**
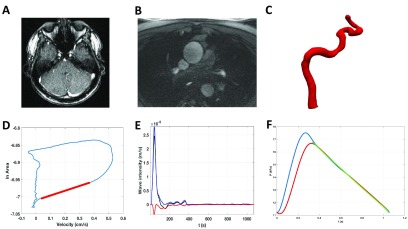
Methods of analysis. (A) Phase contrast imaging of the ICA, (B) phase contrast imaging of the aorta, (C) template derived for the statistical shape modelling, (D) example of lnA-U loop for the estimation of wave speed (gradient as indicated in red), (E) wave intensity (m s^−1^) net wave energy in black, forward energy in blue and backward energy in red. (F) Example of the reservoir analysis, in blue is the pressure wave calculated from the measured variation in cross-sectional area, in red the reservoir pressure and in green the diastolic decay where the measured pressure and reservoir pressure are identical.

ECG-gated phase contrast MR angiography was acquired in the internal carotid arteries. This was done in the transverse plane perpendicular to the internal carotid arteries at the level of the basilar artery (figure 1(A)). Flow data was also acquired in the axial plane for the ascending aorta at the level of the main pulmonary trunk (figure 1(B)). The flows were performed during free breathing. The acquisition parameters of the phase contrast MR angiography (PC-MRA) were as follows: field of view  =  250 mm, slice thickness  =  5 mm, matrix  =  256  ×  100, voxel  =1.0  ×  1.0  ×  5 mm, TR  =  10.95 ms, TE  =  2.61 ms, phase reconstructions  =  100, maximum velocity encoding (VENC)  =  150 cm s^−1^.

### Segmentation

The right ICA and ascending aorta PC-MRA datasets were manually segmented using the magnitude image (Argus, Siemens Healthineers, Erlangen, Germany). The average blood flow velocity (*U*) was calculated automatically in each frame over the cross-sectional area. The segmentations were also used for the calculation of cross-sectional area (*A*) throughout the cardiac cycle. The anatomy of the ICA was reconstructed, meshed and smoothed in its entirety (i.e. from the cervical section of the ICA to the point of entry into the circle of Willis) from the ToF angiograms as a 3D model using 3D slicer (www.slicer.org/; Fedorov *et al* 2012) as shown in figure 1(C).

### Wave intensity analysis (WIA)

The *A* and *U* data were smoothed by the application of a Savitsky–Golay filter with a window size of 11 and a polynomial order of 2. Following the WIA approach using log(area) (Biglino *et al* 2012), the *U*  −  ln*A* loop was plotted and the linear portion of the slope selected during early systole to calculate wave speed (c) by the equation *c*  =  d*U*/dln*A*. The net wave intensity, d*I*  =  d*U*dln*A*, as well as the forward and backward wave components were fitted. Post-processing in MATLAB (The MathWorks Inc., Natick, MA, USA) was performed to identify the peaks and area under the curve of the FCW, FEW, and backward compression wave (BCW).

Wave intensity defined as d*I*_(A)_  =  d*U*dln*A* differs from the original definition of wave intensity, d*I*_(P)_  =  d*P*d*U* as evidenced by their different dimensions. That is, wave intensity d*I*_(P)_ has the units of W m^−2^ whereas the units of d*I*_(A)_ has the units m s^−1^ (Biglino *et al* 2012). However, it can be shown that they differ only by a scaling factor *ρc*^2^, where *ρ*  =  blood density; multiplying wave intensity d*I*_(A)_ by this factor makes it equivalent to the wave intensity originally defined as d*P*d*U*. Here, we have based our comparisons directly on d*I*_(A)_.

### Reservoir analysis

The smoothed area signal (as above) was used to calculate the diameter of the vessel. Using the linear approximation of the relationship between pressure and diameter, the diameter was converted into a pressure estimate using brachial pressure measured during MR acquisition. The reservoir and excess pressure were calculated following the algorithm (Parker *et al* 2012). The diastolic decay (*P*_*∞*_) and time constant (*τ*) was derived from the same algorithm. An example of the reservoir pressure is shown in figure 1(F).

### Statistical shape modelling

The anatomical features of the ICA and its major variation across the study population were assessed by statistical shape modelling (Bruse *et al* 2016). The anatomy of the ICA was modelled from the ToF angiograms described above (see *Image acquisition*). First, the ICAs were manually aligned in 3D space with respect to the cervical ICA as the origin of the alignment. The anatomical mean shape (or ‘template’) was then computed in Deformetrica (www.deformetrica.org, Durrleman *et al* 2014). By comparing each of the ICAs to the mean shape, the variation around the mean was used to compute modes representing the major deformations from the mean carotid shape. This was done according to a previously described methodology (Bruse *et al* 2016). The deformations are divided into a range of modes. Each subject has an associated shape vector for each mode describing deformation in 3D space, e.g. mode 4 describes primarily the tortuosity of the carotid siphon (see figure 5(B)). By assessing the cumulative inertia of the modes, the initial 10 modes were found to describe 95% of the variance observed in the population and therefore the analysis was based on these first 10 modes.

### Statistical analysis

Normality of the data was assessed by D’Agostino-Pearson test of normality. The measures that did not initially pass the normality test were transformed by a log_10_ transformation. Mean differences across the groups were analysed using a one-way analysis of variance (ANOVA) and compared *post hoc* using Dunnet’s multiple comparisons with respect to normotensive values. Correlations were assessed by Pearson’s correlation coefficient. For the shape vectors, both the vector and the magnitude of the vector (without direction) were analysed. Data are reported as mean and standard error of the mean. Alpha was set at 0.05. In the figures, the asterisk signifies the following: ^*^p  <  0.05, ^**^p  <  0.01, ^***^p  <  0.001. The analysis was carried out in GraphPad Prism (GraphPad Prism v6, La Jolla, CA, USA).

## Results

### Demographics

The participants were age-matched within  ±4 years of age. Participants’ demographics are reported in table 1.

**Table 1.** Demographics of the participants.

Variable	NTN	uHTN	cHTN
*n*	13	12	13
Age (years)	55 ± 7.1	58 ± 9.7	56 ± 9.7
BMI (Kg m^−2^)	26 ± 2.1	27 ± 3.5	28 ± 2.5
Ambulatory daytime SBP (mmHg)	122 ± 7.7	143 ± 5.6	127 ± 8.2
Ambulatory daytime DBP (mmHg)	79 ± 7.0	88 ± 7.9	79 ± 6.5
Male:Female ratio	7:5	6:6	4:8
Medications			
– ACEinhibitor	0	5	6
– ATII-inhibitor	0	1	2
– Alpha1-blocker	0	1	0
– Beta-blocker	0	1	0
– Ca^2+^-channel inhibitor	0	3	5
– Diuretics	0	2	2
– No antihypertensive	12	5	0

NTN: normotensive, uHTN: uncontrolled hypertensives, cHTN: controlled hypertensives, BMI: body mass index (Kg m^−2^), SBP: systolic blood pressure, DBP: diastolic blood pressure, ACEinhibitor: acetylcholinesterase inhibitor.

### Wave speed

**Figure 2.**
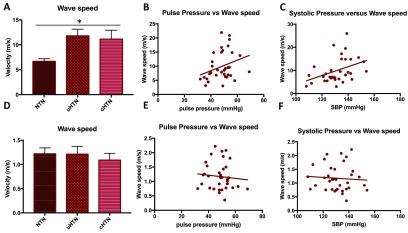
Wave speed (m s^−1^) (A) wave speed (m s^−1^) in the normotensive (NTN), uncontrolled hypertensive (uHTN) and controlled hypertensive (cHTN) groups in the ascending aorta (*p*  =  0.02). (B) Correlation between pulse pressure and wave speed in the aorta (*r*  =  0.3, *p*  =  0.04), (C) correlation between systolic blood pressure and wave speed in the aorta (*r*  =  0.4, *p*  =  0.01), (D) wave speed (m s^−1^) in the NTN, uHTN and cHTN groups in the ICA (*p*  =  0.8), (E) correlation between pulse pressure and wave speed in the ICA (*r*  =  −0.09, *p*  =  0.6), (F) correlation between systolic blood pressure and wave speed in the ICA (*r*  =  −0.07, *p*  =  0.7). Data presented are mean  ±  SEM.

Wave speed in the aorta was significantly lower in the normotensive group (6.7  ±  1.8 m s^−1^, ANOVA *p*  =  0.0197, see figure 2(A)) compared to both the uncontrolled (11.8  ±  4.6 m s^−1^, *p*  =  0.02 Dunnett *post hoc*) and controlled hypertensive (11.2  ±  6.2 m s^−1^, *p*  =  0.04 Dunnett *post hoc*). The wave speed in the ICA was not significantly different between the groups (figure 2(D)).

Wave speed in the aorta was positively correlated with ambulatory daytime pulse pressure (*r*  =  0.3, *r*^2^  =  0.09, *p*  =  0.04, figure 2(B)) and systolic blood pressure (*r*  =  0.4, *r*^2^  =  0.2, *p*  =  0.01, figure 2(C)), however this correlation was not present in the ICA (figures 2(E) and (F)). Wave speed in the ICA negatively correlated with age (*r*  =  −0.4, *r*^2^  =  0.13, *p*  =  0.03). The wave speed calculated in the ICA for all participants was unexpectedly low in comparison to the wave speed measured in other systemic arteries of similar calibre. This is discussed further in the ‘Discussion’.

### Wave intensity

**Figure 3.**
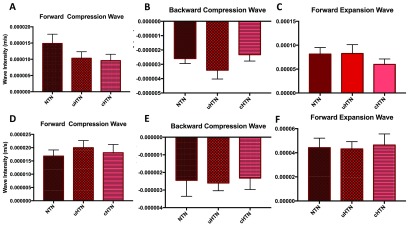
Wave intensity (m s^−1^) of (A) the FCW in the aorta in each of the three groups, (B) backwards compression wave in the aorta, (C) FEW in the aorta, (D) FCW in the ICA, (E) BCW in the ICA, (F) FEW in the ICA. Data presented are means  ±  SEM.

There were no statistical differences between the groups for the net wave intensity, the forward compression or expansion waves, nor for the backward wave intensity or energy in the aorta or in the ICA (see figure 3). There was a significant correlation between age and net forward energy (*r*  =  −0.5, *r*^2^  =  0.3, *p*  =  0.0015), and age and the FEW (*r*  =  −0.6, *r*^2^  =  0.3, *p*  =  0.0002) in the aorta, but not in the ICA.

Despite not reaching statistical significance, we observed a trend for the FCW to be smaller in hypertensive compared to the normotensive volunteers in the ICA. Furthermore, the BCW appeared larger in the uncontrolled hypertensives as compared to both the normotensive and controlled hypertensive groups.

### Reservoir pressure

**Figure 4.**
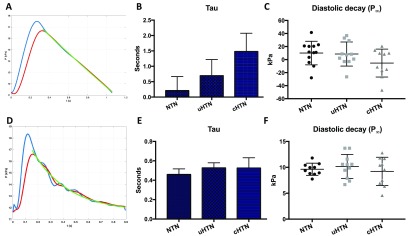
Reservoir and excess pressure in the aorta (A)–(C) and ICA (D)–(F). (A) Example of reservoir pressure (red), diameter-derived pressure (blue) and diastolic decay (green) in the aorta, (B) the time constant, tau, in the aorta for each of the three groups, (C) the diastolic decay (*P*_∞_) for each of the three groups in the aorta, (D) example of reservoir pressure (red), diameter-derived pressure (blue) and diastolic time constant (green) in the ICA, (E) the time constant, tau in the ICA for each of the three groups, (F) the diastolic decay (*P*_∞_) for each of the three groups in the ICA. Tau is presented are means  ±  SEM. The diastolic decays (*P*_∞_) are presented as means  ±  SD.

Reservoir analysis from MR data was feasible, both in the aorta and the ICA. There was no statistical difference between the groups for the mean reservoir, diastolic time constant (*p*  =  0.09, figures 4(A)–(C)) or the excess pressure (*p*  =  0.16) in the aorta. There was also no difference in the reservoir pressure (*p*  =  0.79) or excess (*p*  =  0.37) in the ICA (figures 4(D) and (E)). Reservoir pressure was a positive correlation with systolic blood pressure (*r*  =  −0.4, *r*^2^  =  0.1, *p*  =  0.03) and mean arterial pressure (*r*  =  −0.3, *r*^2^  =  0.1, *p*  =  0.048) in the aorta, but not in the ICA. There was no significant difference in the value of *τ* across the groups in the aorta (*p*  =  0.26) and ICA (*p*  =  0.77). Although it was noted that *τ* was substantially lower in the NTN group, where also wave speed was lower, yet no difference was observed between the uHTN and cHTN.

### Statistical shape modelling

**Figure 5.**
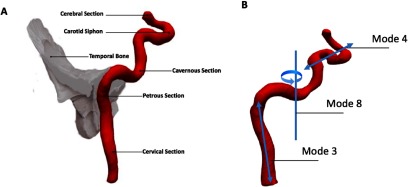
Modes of the statistical shape analysis. (A) Anatomy of the ICA, (B) examples of the modes describing specific features of the shape/anatomy of the ICA; e.g. mode 3 refers primarily to the straightness of the cervical section of the ICA, mode 4 primarily to the tortuosity of the carotid siphon and mode 8 primarily to the rotation of the ICA around the midpoint.

**Figure 6.**
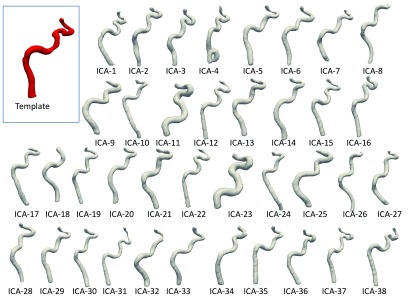
The 3D reconstructed ICAs shown in grey and the template shown in red.

Each shape mode was examined to understand the morphological feature(s) each mode contributed to the shape, e.g. size of the vessel, and tortuosity across the carotid siphon. The modes of greatest interest for the subsequent analysis are presented in figure 5. The first ten modes (recapitulating almost 95% of shape variability in the population) are summarised in table 2. The reconstructed shapes for each individual and the template is shown in figure 6.

**Table 2.** Mean shape features.

Mode	Inertia (%)	Total (%)	Mean Shape feature
1	34.6	34.6	Size of the artery
2	15.2	49.8	Tortuosity of the cervical section of the ICA
3	12.6	62.5	Lateral displacement of the cervical section of the ICA
4	10.1	72.6	Tortuosity across the carotid siphon
5	7.0	79.6	Superior/inferior displacement of the petrosal bend
6	6.1	85.7	Tortuosity of the cavernous section of the ICA
7	3.2	88.9	Rotation and lateral displacement of the carotid siphon
8	2.7	91.6	Rotation over a midpoint between the petrosal bends
9	1.8	93.4	Cranio-caudal stretch
10	1.8	95.2	Axial rotation with respect to the cranio-caudal stretch (mode 9)

*Note*: Each mode presented with the cumulative inertia, the total inertia, and the main describing shape feature.

There was no overall significant difference between the groups when looking at the shape vectors for each of the modes. Interestingly, there was a significant correlation between the FCW and mode 1 which represents the size of the artery (*r*  =  −0.4, *r*^2^  =  −0.12, *p*  =  0.04). Age was significantly correlated with mode 3, which represents the straightness of the cervical ICA (*r*  =  −0.6, *r*^2^  =  0.32, *p*  =  0.004). Flow (l/min) in the ICA also correlated with mode 1 (*r*  =  −0.6, *r*^2^  =  0.31, *p*  <  0.001) and mode 3, the straightness of the cervical ICA (*r*  =  0.4, *r*^2^  =  0.18, *p*  =  0.01). Mode 8, which describes lateral displacement of the cervical ICA and the siphon with respect to the midpoint between the 1st and 2nd petrosal bend, correlated with 24 h ambulatory MAP (*r*  =  −0.3, *r*^2^  =  0.12, *p*  =  0.04) and with DBP (*r*  =  −0.3, *r*^2^  =  0.20, *p*  =  0.007).

For some modes only, it is valid to look at the magnitude of the vector without considering its direction; that is to purely explore the overall distance of the variation from the template regardless of the direction of the displacement. Looking at the magnitude of the vector for mode 10, the axial rotation with respect to the poles of the vessel was positively correlated with 24 h ambulatory pulse pressure (*r*  =  0.4, *r*^2^  =  0.15, *p*  =  0.021) and SBP (*r*  =  0.4, *r*^2^  =  0.17, *p*  =  0.014). DBP also showed a positive correlation with the magnitude of the vector for mode 5 representing the curvature of the 1st and 2nd petrosal bends (*r*  =  0.4, *r*^2^  =  0.19, *p*  =  0.01).

## Discussion

This study describes the feasibility of wave intensity, reservoir pressure and statistical shape analysis in the ICA based on MR data. At present, no *in vivo* study has looked at wave intensity changes in the ICA, in part due to the complex position of the artery and difficulty of measuring pressure, velocity or area given the encased anatomical position. The wave intensity and reservoir pressure measures were also assessed in the aorta to verify that the outcomes of the algorithms are consistent with current literature.

As expected, wave speed in the aorta positively correlated with pulse pressure. Whilst an increased wave speed was expected in the uncontrolled hypertensives given the increased pressure, it is interesting that the wave speed was also increased in the controlled hypertensives. This may suggest that a remodelling of the arterial wall has occurred, which persists after the successful pharmacological control of pressure. Comparing the three groups suggests that elevated wave speed due to hypertension (treated or untreated) accounts for 12.2–6.7  =  5.5 m s^−1^ while pressure in the untreated hypertensives accounts for only for ~1/10th of the increased wave speed, i.e. 11.8–11.2  =  0.6 m s^−1^. Unfortunately, we did not have the power to deduce the effect of different drug classes on wave speed nor collect information on the duration of treatment or diagnosis. Nonetheless, the results are intriguing and warrant further investigation.

However, this relationship was not observed in the ICA. It is well-established that an increased wave speed is indicative of increased stiffness of the vessel. The calculated wave speed in the ICA is much lower than that found in other systemic arteries of similar size. It is also lower than wave speeds reported from simulation data in the common carotid artery (Aguado-Sierra *et al* 2008). It cannot be ruled out that the lower wave speed in the ICA is due to the inherent and inevitable reflections of the wave in the ICA due to the anatomical position between the circle of Willis, representing a strong reflection point, and the petrosal bone, representing a strong anatomical barrier for re-reflections. This is further enforced by the isovolumetric constraints on the intracranial circulation, imposing a unique limitation on the arterial wave mechanics, which have not been studied in any depth. The calculation of wave speed from the *U*  −  ln*A* loop presumes that backward reflections are minimal during early systole, which may not be the case in the ICA. However, the lack of correlation between pressure and wave speed in the ICA could reflect a separate mechanism of adaptation in the ICA to protect against the increased pressure. For example, an increase in the tortuosity of the ICA could act as a buffer for the pressure wave and slow the wave speed before entering the circle of Willis.

The lack of correlation between wave speed and pulse pressure in the ICA was surprising. Given the anatomical position, a larger reflected wave in the ICA was expected given the proximity of the measurement site to the circle of Willis, acting as a major reflection site. However, as a relatively small BCW was observed, it remains to be understood whether this is due to the inherent limitations of the analysis (discussed further below) or a feature of the cerebral feeding vessels physiology.

No significant differences were observed in the FCW, BCW or FEW in the aorta or ICA between the groups. However, there was a trend for the FCW to be higher in the NTN volunteers (1.5  ×  10^−5^ m s^−1^) compared to the uHTN (1.0  ×  10^−5^ m s^−1^) and cHTN (9.6  ×  10^−6^ m s^−1^) in the aorta. In particular, the cHTN showed a trend toward a lower FCW compared to the normotensives. Interestingly, the opposite trend was true for the ICA in which FCW tended to be lower in the NTN group (1.7  ×  10^−5^ m s^−1^) compared to that of the uHTN and cHTN (2.0  ×  10^−5^ and 1.8  ×  10^−5^ m s^−1^, respectively).

The estimation of reservoir and excess pressures was feasible from MR data, as brachial pressure data was also available. A significant relationship between blood pressure, reservoir and excess pressure has been previously reported (Narayan *et al* 2015). This was observed as well in the present study for the aorta, but not the ICA. We found a tendency towards higher values of *τ* in the hypertensive aorta. Given the increased wave speed, a smaller value of *τ* would be expected in the hypertensives. A possible explanation for this unexpected observation may be found in the mechanism of the regulation of cerebral blood flow. It is observed that cerebral blood flow is relatively constant over a wide range of perfusing pressures, presumably through the control of cerebral vascular resistance. If this mechanism were working in hypertension, we would expect the higher resistance to increase the diastolic time constant, which is the product of cerebral vascular resistance and compliance. Whilst the calculation of reservoir pressure was feasible, we observed some non-physiological parameters in the data, most prominently observed as a negative diastolic decay (*P*_∞_) in the aorta. This was due to a concave fall-off in the diastolic area data, which is not considered physiological. However, this is most likely due to the nature of the acquisition of our MR data. Given that the data is averaged over ~5 min of acquisition, the variability in the cardiac cycle (e.g. due to natural variation such as sinus arrhythmia) will be most pronounced in diastole given that there is little to no change in the PT interval compared to TP interval under resting conditions and the ECG is used to trigger the acquisition. This will inevitably lead to more variability in the data around the TP segment of the ECG and therefore a less defined edge of the diastolic wall compared to the edge detection in systole. Considering the shape of the area data derived from PC-MR images compared to the shape of a flow or pressure wave suggests that the area may provide additional information regarding the haemodynamic wave discharge in diastole. This is an intriguing observation that warrants further research.

Further, the reservoir pressure was based on the linear conversion of diameter to pressure by means of brachial pressure. While there is support for the linearity between brachial pressure and carotid pressure (Willemet and Alastruey 2015), this assumption is based on *in silico* data. There are currently no studies available on the presence or absence of the reservoir effect in the ICA. Whilst this remains a point for future investigation, our data suggest the presence of the reservoir effect in ICA, and further suggest the feasibility of using systemic pressure to estimate the size of the carotid pressure components. Nonetheless, we acknowledge the limitations of the pressure measured by sphygmomanometry at the brachial artery are two-fold. Firstly, the pressure in the aorta and internal carotid arteries may not be the same as that of the brachial artery; this effect could be controlled for in future studies if the pressure in the internal carotid could be estimated. However, at present we were not able to identify a non-invasive mechanism for measuring or estimating the ICA pressure accurately. Secondly, the sphygmomanometric measure provides only systolic and diastolic pressures and does not record the pulse waveform. If the pulse waveform had been recorded either prior to the scan or by proxy via either a collected ECG trace during the scan or finometry recorded outside the scanner, this waveform could be superimposed onto the recorded area data to aid in the identification of crucial time points such as the dicrotic notch. This would lend further confidence to the accuracy of the estimation.

It is interesting that we observed a correlation between the FCW and the size of the artery (mode 1) as part of the statistical shape analysis, and further pressure data (i.e. MAP, SBP and DBP) correlated with anatomical features of the ICA. On top of showing the feasibility of applying a statistical shape modelling framework to study smaller vessels and particularly the cerebral circulation, this may also suggest that the complex shape of the ICA contributes to the wave dynamics, and in turn that this may be influenced by blood pressure. Further investigation into the relationship between arterial wave dynamics and arterial shape morphology is warranted.

### Limitations

The current study aimed to explore the feasibility of deriving wave intensities and reservoir pressure in the ICA from MRI data. Whilst our MR data for both the aorta and ICA have a high spatial (~1  ×  1 mm) and temporal resolution (effective temporal resolution ~10 ms), the data suffers from some movement artefacts due to the necessity to acquire the images during free breathing (and free swallowing). Acquiring the data during a breath-hold to avoid movement would currently imply compromising on the temporal resolution. Furthermore, studying medium and small diameter arteries such as the ICA (~0.2 cm in diameter) is complicated by the very small changes in cross sectional area. Whilst current software allows for the segmentation of the arteries, the spatial resolution achievable means that the changes are more susceptible to noise compared to bigger vessels such as the aorta. This is due to partial volume effects of a curving structure defined digitally by square pixels.

### Conclusion

It is feasible to study wave dynamics non-invasively in the ICA *in vivo* using high-resolution CMR imaging. Observations on the effect of vessel morphology on hydrodynamic and energy parameters should be explored in larger populations.
